# Improvements in thoracic surgery outcomes: a multi-institutional collaboration study

**DOI:** 10.1186/s13019-015-0228-7

**Published:** 2015-03-06

**Authors:** Yasushi Iwasaki, Junichi Shimada, Daishiro Kato, Motohiro Nishimura, Kazuhiro Ito, Kunihiko Terauchi, Masanori Shimomura, Hiroaki Tsunezuka

**Affiliations:** 1 Department of Thoracic Surgery, Nantan General Hospital, Kyoto, Japan; 2 Division of Chest Surgery, Department of Surgery, Graduate School of Medical Science, Kyoto Prefectural University of Medicine, Kyoto, Japan; 3 Department of Thoracic Surgery, Saiseikai Suita Hospital, Osaka, Japan; 4 Department of Thoracic Surgery, Yamashiro General Medical Center, Kyoto, Japan; 5 Department of Chest Surgery, Nara City Hospital, Nara, Japan; 6 Department of Thoracic Surgery, Ayabe City Hospital, Kyoto, Japan

**Keywords:** Thoracic surgery, Standardization, Operating room nursing, Organizational efficiency, Safety management

## Abstract

**Background:**

Treatment protocols (including those for thoracic surgery) tend to be customized for individual hospitals. Procedural standardization is required to improve surgical tasks and patient outcomes. This study aimed to evaluate the effects of an initiative to standardize surgical tasks for efficient and safe performance.

**Methods:**

Hospitals associated with the Division of Chest Surgery of the Kyoto Prefectural University of Medicine held joint meetings involving their thoracic surgeons and operating room nurses between February 2011 and November 2012 to standardize surgical tasks. Operation times and blood loss were compared before and after standardization.

**Results:**

The implementation rate of standardized surgical tasks was 97%. The pre-operative (from entry to the operating room until commencement of surgery) and post-operative (from conclusion of surgery until departure from the operating room) times were significantly decreased after the standardization. When compared according to operative group (all thoracic surgery, lung lobectomy, and partial lung resection), operation times were shorter for all three groups; in addition, the amount of blood loss was lower in all three groups after standardization. A post-standardization survey showed improved morale among the meeting participants.

**Conclusions:**

Interdisciplinary standardization of surgical tasks across institutions improved thoracic surgery tasks and surgical outcomes.

## Background

In Japan, lung cancer has been rising in incidence, increasing from 97,000 in 2008 to an estimated 124,000 in 2020; this has been accompanied by increased morbidity [1]. Coupled with an anticipated increase in metastatic lung tumors, this may result in an increasing number of surgeries to treat these conditions [2]. According to the 2011 Annual Report by the Japanese Association for Thoracic Surgery, of the 69,223 chest surgeries performed in 2011, 49% (33,878) were for lung cancer, and surgery for metastatic lung tumors accounted for 10.4% (7,210) [1]. Moreover, because adequate control of primary lesions is now possible, it is anticipated that the number of people undergoing surgery for metastatic lung tumors will continue to rise, rendering thoracic surgery of even greater importance; indeed, thoracoscopic surgery now accounts for a high proportion of operations and is increasing annually [1]. Therefore, surgery is becoming more complex, especially given the remarkable advances in surgical devices such as endoscopic staplers and energy devices [3]. Even though endoscopic surgery is considered minimally invasive, safety concerns [4] regarding blood loss and other problems assume greater importance over the longer time required compared with open surgery. Thus, a high level of knowledge and skills are now required not only of thoracic surgeons but also operating room nurses.

The issue of nursing standards was raised in the FY 2006 revisions to medical treatment service fees, exposing a nationwide shortage of nurses [5]. As a result, the streamlining of all medical tasks is now an urgent issue. At the same time, all hospitals affiliated with universities have site-specific procedures for surgical tasks, following their respective traditions.

The International Organization for Standardization (ISO) and International Electrotechnical Commission (IEC) define “standardization” as an established activity with regard to actual or potential problems and provisions for common and repeated use, aimed at achieving an optimal degree of order in a given context. In particular, the activity consists of the processes of formulating, issuing, and implementing standards [6]. Although standardization has already facilitated considerable progress in industrial fields, its adoption in healthcare has been slow. We hypothesized that the standardization of surgical tasks and streamlining of the entire workflow would result in similar, high-quality medical treatment across different hospitals.

Between February 2011 and November 2012, thoracic surgeons and operating room nurses of the Division of Chest Surgery of the Kyoto Prefectural University of Medicine and associated hospitals participated in an initiative to standardize surgical tasks to ensure that these tasks were performed efficiently and safely. We hereby report the results of this initiative.

## Methods

Surgical tasks were standardized by the Division of Chest Surgery of the Kyoto Prefectural University of Medicine and four associated thoracic surgery hospitals. Joint meetings were held with thoracic surgeons and operating room nurses from all five hospitals to discuss individual surgical tasks on the basis of surveys previously conducted.

The surveys collected data regarding how the tasks were performed in each hospital. In practical terms, we defined the tasks that needed to be standardized as those tasks performed during the period from the patient’s entry into the operating room to the commencement of surgery (pre-operative time) in addition to the period from the conclusion of surgery to the departure from the operating room (post-operative time), as follows: (1) organization of surgical preparation items and introduction of the cart into the operating room; (2) the arrangement of surgical instruments in the operating room; (3) signing patients in upon entry to confirm their identity; (4) time-out before surgery (confirmation of patient’s identity, surgical site, and surgical procedures); (5) use of compression stockings to prevent deep-vein thrombosis and use of a foot pump until patients start walking; (6) patient positioning and immobilization; (7) timing of disinfection and prevention of disinfectant-induced chemical burns; (8) management of body temperature with a warm-air temperature management unit; (9) surgical techniques; (10) presence of surgical instruments (i.e., thoracoscopic systems, endoscopic staplers, energy devices); (11) organization of surgical devices and consistency of nomenclature; (12) installation of a low-pressure suction device routinely; and (13) post-operative thoracic radiography and its timing.

After the meetings, a surgical task manual was prepared, including video footage of surgical techniques; this was distributed to all participating hospitals in a DVD format to ensure that staff members were informed of the new standards.

A survey was carried out at each hospital one month after the series of joint meetings to assess how well the surgical tasks had been standardized. Tasks that had been successfully standardized and those that had yet to be standardized were counted separately to determine the standardization rate, which was defined as the number of tasks successfully standardized at each hospital divided by the total number of tasks to be standardized.

The effect of the initiative to standardize surgical tasks and perform operating room tasks efficiently was evaluated by measuring pre-operative time and post-operative time for 10 consecutive patients in each hospital (total: 50 patients) both before and after standardization. To assess the clinical effects of the initiative to standardize surgical tasks, the operation time and amount of blood loss during operations the year before and after standardization at each hospital were also examined.

Based on the patient characteristics (Table 1), patients were divided into three groups: all thoracic surgery, lung lobectomy, and partial lung resection groups; separate results were generated for each group. In the lung lobectomy group, we removed a single lung lobe due to lung cancer. Patients who underwent bronchoplasty or combined resection and those with an intra-operative diagnosis were excluded from the lung lobectomy group.

**Table 1:** **Characteristics of all thoracic surgery patients at all institutions the year before and after standardization**

	Before	After
All chest surgery patients	n=373	n=372
Age	62.0 (14–92)	63.0 (16–93)
Men	244 (65%)	242 (65%)
Disease		
Primary lung cancer	159	189
Metastatic lung tumor	57	66
Benign lung tumor	2	2
Inflammatory lung disease	20	19
Mediastinal tumor	29	25
Chest wall tumor	3	4
Empyema	12	2
Spontaneous pneumothorax	75	60
Cystic lung disease	2	0
Others	14	5
Type of surgery		
Pneumonectomy	3	1
Lobectomy	99	100
Segmentectomy	21	20
Partial lung resection	188	211
Mediastinal tumor removal	26	25
Chest wall tumor resection	4	4
Empyema	12	2
Biopsy	7	3
Others	13	6
Surgical approach		
VATS	69	53
Minithoracotomy (<8 cm)	238	280
Others	66	39

VATS: video-assisted thoracic surgery using only monitor view.

Four months after the joint meetings, a questionnaire was conducted to survey the participants’ change of attitude toward the ideas for improvement of surgical tasks and thoracic surgery.

The data were analyzed using JMP software (SAS Institute, Cary, NC, USA). Wilcoxon ranks-sum tests were used for comparisons between two groups, with the significance level set at *p* < 0.05.

## Results

Overall, standardization was achieved (standardization rate, 97%). Some hospitals had not achieved standardization for time-outs or the timing of disinfection and hand washing.

**Figure 1:**
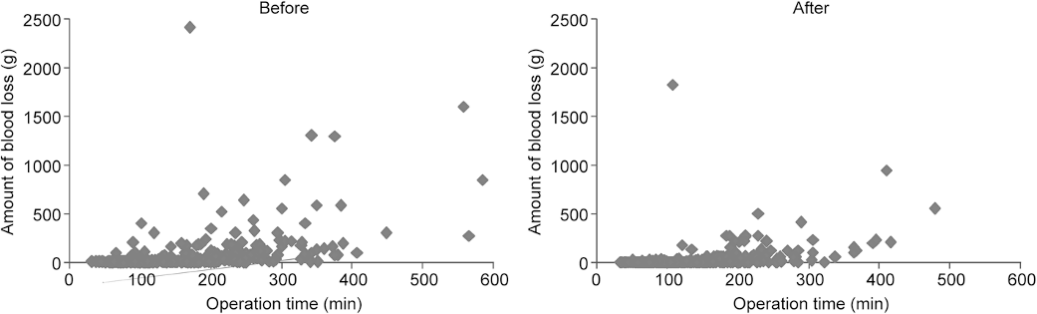
**Scatterplots of the operation time and amount of blood loss before and after standardization of operating room procedures.** The operation time and amount of blood loss are plotted for each patient who underwent thoracic surgery in any of the institutions the year before and after standardization.

Pre-operative and post-operative times significantly decreased, from a median of 59 min to 53 min and 52 min to 38 min, respectively (Table 2). In addition, a significant decrease in operation time was observed in all three groups following the standardization: all thoracic surgery group, 146 min to 116 min; lung lobectomy group, 228 min to 176 min; and lung resection group, 103 min to 92 min (Table 3 and Figure 1). The median amount of operative blood loss also decreased significantly in all three groups: all thoracic surgery group, 10 g to 5 g; lung lobectomy group, 39 g to 10 g; and partial lung resection group, 5 g to 3 g (Table 4 and Figure 1).

**Table 2:** **Pre-and post-operative times for 10 consecutive patients at each hospital (total: 50 patients) before and after standardization of operating room procedures**

	Before n=50	After n=50	p-value
Pre-operative time (min)			
Median (25%, 75%)	59 (52, 70)	53 (48, 61)	0.008
Post-operative time (min)			
Median (25%, 75%)	52 (40, 70)	38 (29, 43)	<0.0001

**Table 3:** **Operation time for thoracic surgery patients in all institutions the year before and after standardization of operating room procedures**

	Before	After	p-value
All cases (min) before: n=373, after: n=372			
Median (25, 75%)	146 (95, 219)	116 (80, 174)	<0.0001
Lobectomy (min) before: n=74, after: n=83			
Median (25, 75%)	228 (192, 262)	176 (145, 213)	<0.0001
Partial lung resection (min) before: n=183, after: n=205			
Median (25, 75%)	103 (76, 144)	92 (74, 116)	0.006

**Table 4:** **Amount of blood loss for thoracic surgery patients at all institutions the year before and after standardization**

	Before	After	p-value
All cases (g) before: n=373, after: n=372			
Median (25, 75%)	10 (3, 56)	5 (3, 12)	<0.0001
Lobectomy (g) before: n=74, after: n=83			
Median (25, 75%)	39 (10, 80)	10 (5, 30)	0.0002
Partial resection (g) before: n=183, after: n=205			
Median (25, 75%)	5 (3, 10)	3 (3, 5)	0.04

**Figure 2:**
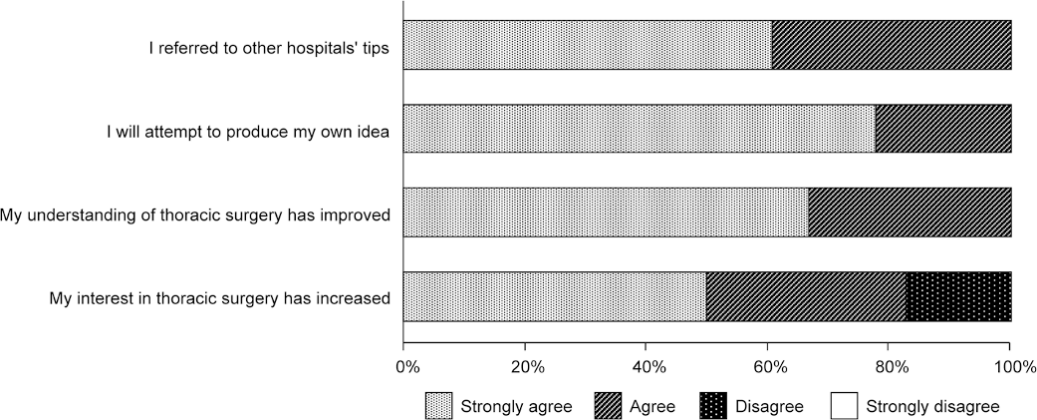
**A post-standardization survey about the change of attitude toward the ideas for improvement of surgical tasks and thoracic surgery for participants of the joint meetings.**

In the questionnaire, all members (n=18) referred to tips for improvement from other hospitals, and they attempted generate their own ideas. All members confirmed improved understanding of thoracic surgery and 83% of the participants confirmed increased interest in thoracic surgery (Figure 2).

## Discussion

The present initiative to standardize operating room procedures evaluated was successful in reducing both the pre-operative and post-operative times, presumably from the streamlined processes. The amount of time spent under anesthesia was also reduced, not only mitigating the burden on individual patients but also increasing efficiencies in the operating room and potentially lowering medical costs.

The Practical Guidelines for Perioperative Care of the Japanese Association for Operative Medicine recommend that individual hospitals produce surgical manuals and revise them in accordance with context, as required. Thus, this policy requires each hospital to use its own unique manual, a situation that does not achieve standardization to the same level as that in the industrial field.

In the healthcare field, the concept of the “clinical path,” was developed in the US in the late 1980s, with the aim of enhancing interdisciplinary collaboration and reducing the time spent by patients in hospitals [7]; in addition, evidence-based clinical guidelines have been available since the 1990s. These efforts could be regarded as progress towards standardization, given that clinical paths comprise a form of standardization related to treatment schedules in individual hospitals; furthermore, clinical guidelines could be generally regarded as a standardized policy for treating individual diseases and symptoms. In contrast, our initiative was characterized by the intent to engage in the detailed standardization of individual procedures involved in operating room tasks.

Stark *et al.* reported that the standardization of surgical procedures for vaginal hysterectomy resulted in improved outcomes, including shorter operation times [8]. In the present study, operation times were also reduced for all surgery types following the standardization of procedures. In practical terms, the standardization of surgical procedures and organization of surgical instruments in individual hospitals improved the coordination of surgical assistance, contributing to a reduction in operation times and intraoperative blood loss. As a result, efficiencies were gained, and treatment safety was improved.

The participants of the joint meetings were surveyed after the standardization initiative and indicated that their understanding of and interest in thoracic surgery had increased. In addition, tips for improvement from other hospitals stimulated additional ideas, indicating an increase in morale among the medical staff. The involvement of thoracic surgeons and operating room nurses who performed the tasks may have had a considerable effect on morale and the generation of the observed outcomes.

Although the initiative was generally successful, it proved ineffective for some tasks, owing to certain restrictions at individual hospitals. With respect to time-outs, hospital-wide agreements that were applicable to all clinical departments took precedence in some hospitals; these could not be changed purely on the basis of agreements between the Department of Chest Surgery and the operating room. Once again, this suggested that hospital-wide initiatives, as opposed to those for individual clinical departments, may be required to promote standardization.

The content of this initiative was not unique to thoracic surgery; therefore, it could be adapted for surgical tasks in other areas of surgery. The use of similar initiatives could lead to similar results.

## Conclusions

An interdisciplinary initiative to standardize surgical tasks in thoracic surgery across multiple institutions was useful in improving thoracic surgery tasks and boosting morale among the participating surgeons and nurses. This resulted from the exchange of information that took place during the development of the initiative, ultimately leading to improved surgical outcomes.
